# On the Springback and Load in Three-Point Air Bending of the AW-2024 Aluminium Alloy Sheet with AW-1050A Aluminium Cladding

**DOI:** 10.3390/ma16082945

**Published:** 2023-04-07

**Authors:** Stanisław Kut, Grzegorz Pasowicz, Feliks Stachowicz

**Affiliations:** 1 Department of Materials Forming and Processing, Rzeszow University of Technology, al. Powstańców Warszawy 8, 35-959 Rzeszów, Poland; 2 Doctoral School of the Rzeszow University of Technology, al. Powstańców Warszawy 12, 35-959 Rzeszów, Poland

**Keywords:** three-point air bending, bending load, springback, clad sheet metal

## Abstract

This article presents the results of an analysis of the bending load characteristics and the springback phenomenon occurring during three-point bending of 1.0 and 2.0 mm thick AW-2024 aluminium alloy sheets with rolled AW-1050A cladding. A new proprietary equation was proposed for determining the bending angle as a function of deflection, which takes into account the influence of the tool radius and the sheet thickness. The experimentally determined springback and bending load characteristics were compared with the results of numerical modelling using different models: Model I, a 2D model for a plane deformation state, disregarding the material properties of the clad layers; Model II, a 2D model for a plane deformation state, taking into account the material properties of the cladding layers; Model III, a 3D shell model with the Huber–von Mises isotropic plasticity condition; Model IV, a 3D shell model with the Hill anisotropic plasticity condition; and Model V, a 3D shell model with the Barlat anisotropic plasticity condition. The effectiveness of these five tested FEM models in predicting the bending load and springback characteristics was demonstrated. Model II was the most effective in predicting bending load, while Model III was the most effective in predicting the amount of springback after bending.

## 1. Introduction

In many industries, especially in aviation and automotive production, the selection of material is based on the ratio of its density to strength [1]. In the aviation industry, the materials used for aircraft coatings are high-strength aluminium alloys; however, the disadvantage of these materials is their low corrosion resistance. Corrosion damage occurring during operation is a serious threat, especially for aging aluminium aircraft structures [2,3]. A way to improve the corrosion resistance of the materials is to apply protective coatings made of corrosion-resistant materials. In the case of high-strength aluminium alloy sheets, technically pure aluminium is used as a material for coatings, and applied on both sides of the parent material in the rolling process [4].

Bending is one of the processes of shaping sheet metal elements with unfolded surfaces. Regardless of the bending method and operation used, three basic stages can be distinguished in this process, i.e., elastic bending, plastic bending and springback. One of the features determining the quality of bent elements is their dimensional and shape accuracy. Due to this reason, the main problem during the analysis and design of this process is the determination of the amount of springback of the material after bending and the bending load.

**Figure 1:**
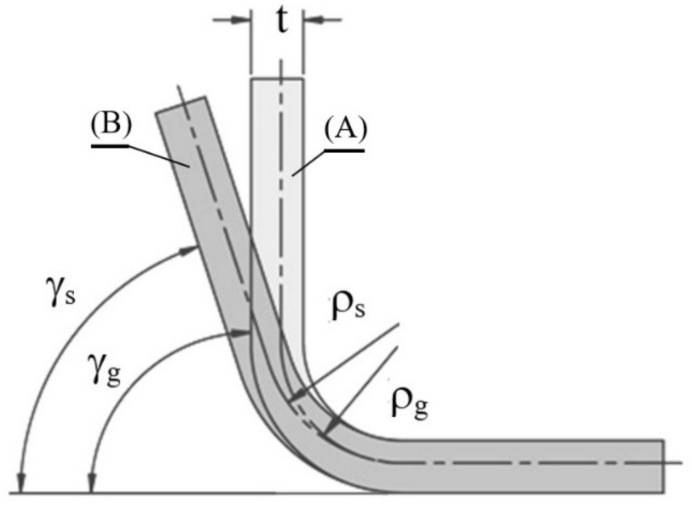
The shape of a bent sample: (**A**) under load and (**B**) after unloading.

An analysis of the bending process can be carried out with the use of analytical, experimental and numerical methods. The springback value is most often determined by the dimensionless springback factor, K. This factor is calculated as the quotient of the bending radius under load, ρ_g_, and the bending radius after unloading, ρ_s_, or as the quotient of the bending angle after unloading, γ_s_, and the bending angle under load, γ_g_ (Figure 1).

(1) $$K=\frac{\gamma_{s}}{\gamma_{g}}=\frac{\rho_{g}}{\rho_{s}}$$

where:

γ_g_ and γ_s_—band deflection angles under load and after springback, respectively,
ρ_g_ and ρ_s_—band radii under load and after springback, respectively.

Knowing the value of this factor for specific bending cases enables calculating the radius or angle that should be used during bending in order to obtain a product of the desired shape after bending. In other words, it makes it possible to eliminate or at least minimize the adverse effects of the springback phenomenon after bending in the process and technological equipment design stage. On the other hand, the knowledge of the course and values of the bending load of components is necessary when designing dies and tools, as well as in the selection of bending presses.

Depending on the accuracy of the results of the analysis of the bending process in the design stage, in engineering practice, the correction of the shape of prototype bending tools specified in the design is often performed by trial and error, with the number of trials decreasing as the accuracy of the calculation result increases. From this aspect, it is extremely important not only to know the computational methods used in this area, but also the potential of these methods and their effectiveness depending on the models used and the degree of inclusion in these models of factors influencing the analysed parameters of the analysed process.

In the scientific literature, one can find many works devoted to analyses of the sheet bending process, including the process force parameters and sheet springback for various materials, taking into account various models of strain hardening, various plasticity conditions, changes in the value of Young’s modulus, the Bauschinger effect and others. Various approaches have been used to solve the issues under consideration, e.g., analytical [5,6] and numerical [7,8], as well as using neural networks [9]. In most numerical calculations related to the bending process, both implicit and explicit methods are used using commercial software. Much less information is available on the effect of cladding layers in predicting the springback or bending load of clad plates. One can find only the results of research on bending of laminated (double layer) sheets, conducted mainly in terms of determining the impact of the thickness of individual layers on the value of the bending load and springback [10] or determining the bendability limit of such materials [11,12]. However, there are no studies available on clad (three-layer) sheets, which is the subject of this study. In a study on the analysis of the bending process of double clad (three-layer) Cu/Al/Cu sheets [13], the focus was mainly on determining the cohesion of individual layers after deformation.

This paper presents the results of experimental research and numerical analyses of the three-point air bending process of AW-2024 aluminium alloy with rolled AW-1050A cladding for anti-corrosion protection which, which, among others, is used to make the outer plating of aircraft structures. The bending load and springback characteristics determined experimentally were compared with those obtained in the process of numerical modelling with the use of various plasticity conditions.

## 2. Material and Experimental Procedure 

**Figure 2:**
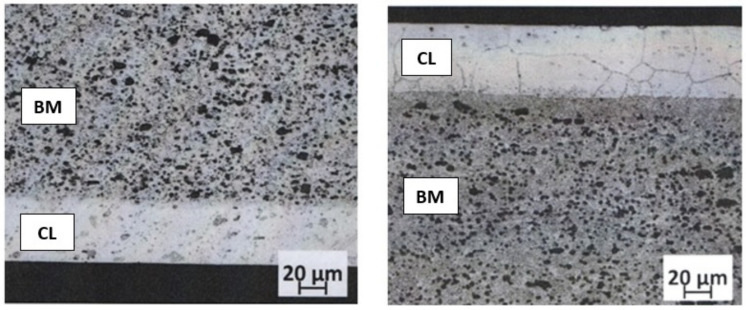
The microstructure of the tested sheets in the cross-section of sheets with a thickness of 1.0 mm (**left**) and 2.0 mm (**right**). CL—cladding layer, BM—base material.

**Figure 3:**
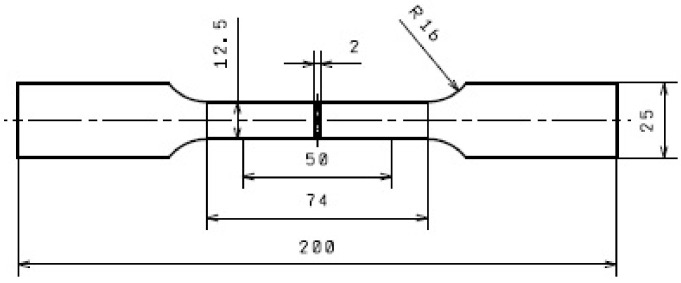
Shape and dimensions of test specimens in mm.

The tests were carried out with the use of AW-2024 aluminium alloy sheets with the thicknesses of 1.0 and 2.0 mm used as-received, i.e., after softening annealing. The chemical composition of the tested sheet material is presented in Table 1. The thickness of the clad layers applied on both sides was 50 µm for a 1.0 mm sheet and 55 µm for a 2.0 mm sheet (Figure 2), which corresponds to 10.0% and 5.5% of the sheet thickness, respectively. The rolled AW-1050A cladding layer closely adheres to the AW-2024 sheet material, with no visible transition zones. From the supplied sheets, samples were taken for uniaxial stretching in the 0°, 45° and 90° directions according to the rolling direction of the sheets. The shape and dimensions of samples for testing in accordance with ISO 6892-1 are shown in Figure 3.

Quasi-static uniaxial tensile tests of individual samples were carried out on a Zwick/Roell Z030 testing machine by measuring the elongation and change in the sample width using a multi extensometer. Three test samples were taken for each of the rolling directions, resulting in nine samples in total for each of the tested sheets. Tensile (and bending) tests were carried out at the room temperature of 293 K. The tensile speed of the samples was 30 mm/min. On the basis of the results of these tests, strain hardening curves were developed. They were used to determine the material parameters for the four models of the strain hardening curve described later in this work and presented in Section 4. The plastic anisotropy factor, r, was determined on the basis of the relationship between the width strain and thickness strain in the whole range of straining using the method proposed by Welch et al. [15]. For both the 1.0 mm thick sheet and the 2.0 mm thick sheet, the value of the yield strength of the base material was more than twice as high as the yield strength of the cladding material, while the value of the normal anisotropy of the cladding material was much higher than the value of this parameter for the base material (Table 2).

For practical reasons, the curves of strain hardening are presented in the form of constitutive equations of the so-called function of flow stress. Such equations were used, among others, for the analysis and simulation of cold forming processes at relatively low strain rates, when their influence on the flow stress can be neglected. In this paper, four models of flow stress of various complexity levels were selected to describe the course of the material strain hardening of the tested sheet:

- Hollomon [16]:

(2) $$\sigma_{p}\left(\varepsilon_{p}\right)=K_{1}\varepsilon_{p}^{n_{1}}$$

- Swift [17]:

(3) $$\sigma_{p}\left(\varepsilon_{p}\right)=K_{2}{\left(\varepsilon_{0}+\varepsilon_{p}\right)}^{n_{2}}$$

- Voce [18]:

(4) $$\sigma_{p}\left(\varepsilon_{p}\right)=A_{3}+K_{3}\left(1-\exp(-n_{3}\varepsilon_{p}\right))$$

- El-Magd [19]:

(5) $$\sigma_{p}\left(\varepsilon_{p}\right)=A_{4}+B_{4}\varepsilon_{p}+K_{4}\left(1-\exp(-n_{4}\varepsilon_{p}\right))$$

where σ_p_—flow stress, ε_p_—equivalent plastic strain, K_1_–K_4_, A_3_, A_4_, B_4_, ε_0_ and n_1_–n_4_—material constants determined experimentally.

The Hollomon model, which is the simplest and most often used in engineering, is the strain hardening model, and provides a good description of the hardening curve in a wide range of deformations, which is why it is commonly used in modelling plastic forming processes, especially those with large deformations, such as forging, extrusion, punching, etc. The Swift model, similar to the Hollomon model, due to its versatility but also greater accuracy in the description of the initial course of the strain hardening curve, is very often used in the numerical modelling of a wide range of plastic forming processes in the field of small and large deformations. The Voce model is also often used to describe the course of the strain hardening curve, which, similar to the Swift model, requires knowledge of three material constants. The most complex of the selected models is the Voce model, extended with an additional linear component, and its use requires determination of as many as four material constants. In the literature, the El-Magd model is also referred to as the extended Voce model [20]. 

**Figure 4:**
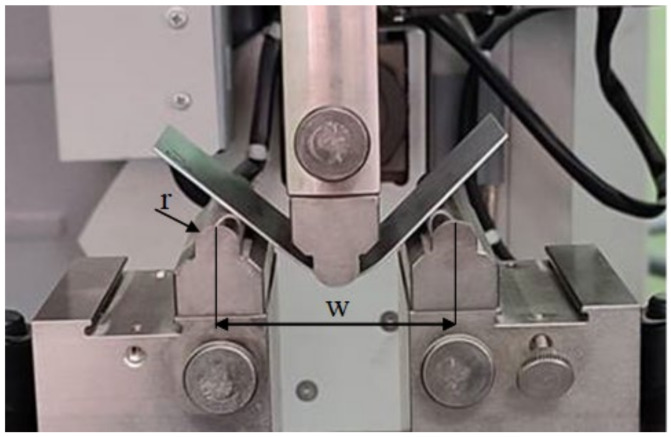
A photograph of a bent sample.

Bending specimens that were 40 mm wide and 100 mm long were taken parallel to the rolling direction. Experimental bending tests were carried out on a Zwick/Roell Z030 testing machine in a three-point tool system with the same radii (r = 5 mm) (Figure 4). For each of the tested sheets, the curves of the bending load as a function of the deflection arrow of the samples were determined in a one load cycle in the range of the deflection arrow (0–20 mm) and in eight load cycles for the deflection, which were successively under load (2, 4, 6, 8, 11, 14, 17 and 20 mm). Then, based on the experimental data, the numerical values of the deflection arrow of the sample after unloading in subsequent bending cycles were determined. The values of the bending angle and the springback angle in subsequent cycles were calculated, together with numerical values of the springback factor corresponding to these cycles. 

To calculate the bending angles, γ_g_, and springback, γ_s_, in individual bending cycles, Equations (6) and (7), respectively, are most often used. However, these formulas do not take into account the radii of the bending tools and the thickness of the bent strand [9].

(6) $$\gamma_{g}=\mathrm{arctg}\left(\frac{f_{g}}{w}\right)$$

(7) $$\gamma_{s}=\mathrm{arctg}\left(\frac{f_{s}}{w}\right)$$

where

- f_g_ and f_s_—band deflection arrows under load and after unloading,
- w—spacing of external bending rollers.

**Figure 5:**
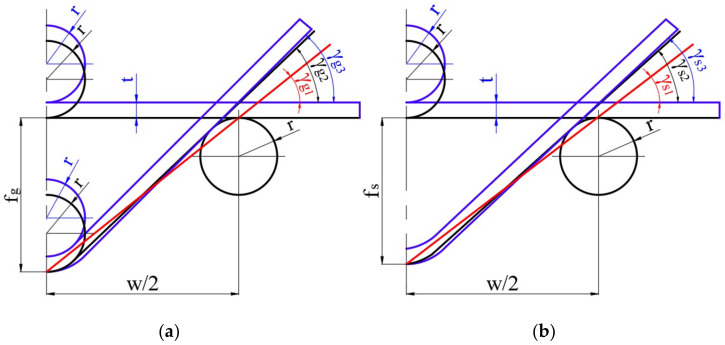
The influence of the geometrical parameters of the model on the value of the bending angle under load, γ_g_, (**a**) and the bending angle after unloading, γ_s_, (**b**). γ_g1_ and γ_s1_— bending angles without taking into account tool radii and sheet thickness, calculated from Equations (6) and (7), γ_g2_ and γ_s2_—bending angles taking into account tool radii without taking into account the sheet thickness, γ_g3_ and γ_s3_—bending angles taking into account tool radii and sheet thickness, calculated from Equations (8) and (9).

Simultaneously, it can be observed that as the deflection arrow grows, the location of the contact point of the sample with the surface of the shaping tools changes (Figure 5), which has a decisive impact on the value of the calculated bending angle (Figure 5a) and springback angle (Figure 5b). Therefore, original formulas were proposed for calculating the angle of bending (Equation (8)) and springback angle (Equation (9)), which take into account the observed phenomenon and also consider the influence of the thickness of the band:

(8) $$\gamma_{g}=\mathrm{arctg}\left(\frac{2f_{g}}{w-{\mathrm{Af}}_{g}}\right)$$

(9) $$\gamma_{s}=\mathrm{arctg}\left(\frac{2f_{s}}{w-{\mathrm{Af}}_{s}}\right)$$

where A—dimensionless coefficient depending on the thinness coefficient, t/r, and the size of the spacing of the external bending rollers, w. The value of parameter A was determined on the basis of geometrical relationships using the least squares method with the use of the Logger Pro program. The data used to determine the value of coefficient A for w = 50 mm and w = 100 mm are shown in Table 3 and Table 4, respectively. The values of the angles γ_g3_ or γ_s3_ for individual deflection arrows f_g_ or f_s_, respectively, were determined using CAD software.

**Figure 6:**
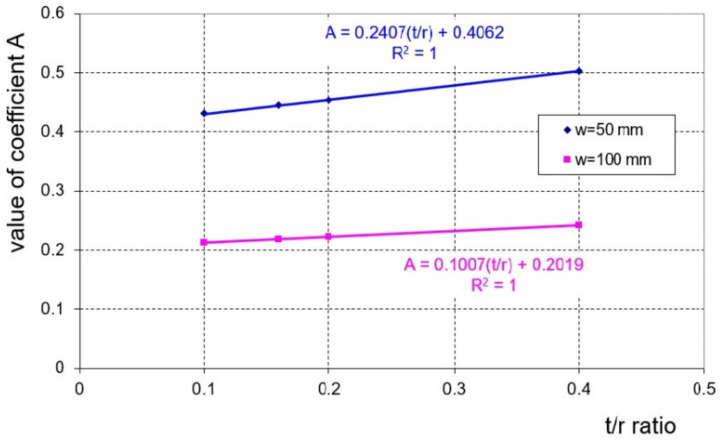
Dependence of the value of coefficient A on the t/r ratio and the size of the spacing of the external bending rollers, w.

The dependence of the value of coefficient A on the value of the ratio of the sheet thickness to the radius of the bending rollers, t/r, is more greatly emphasised with the smaller spacing of the external bending rollers (Figure 6).

## 3. Numerical Modelling

An analysis of the bending process was performed with the use of a nonlinear FEM and the commercial MSC.MARC/Mentat 2020 software. The aim of this analysis was to determine the influence of five various variants of FEM models on the effectiveness of forecasting the springback value and the bending load of clad sheets. The numerical models were developed based on the experiments, taking into account the dimensions of samples and tools as well as the pattern of loading the samples during bending. In all models, the deformed strand of material was defined as a deformable body and the tools of the bending device were defined as perfectly rigid bodies. The friction model was described by Coulomb’s law. The friction coefficient between the sample material and the tools was 0.23 [21]. The El-Magd model [19] was used to describe the course of the strain hardening curves in all numerical models. The evolution of the surface of plasticity as a result of the phenomenon of strain hardening was described using the isotropic model. In the calculations, the associated law of plastic flow of Prandtl–Reuss and the implicit scheme of integration of differential equations over time with the Newton–Raphson method were used. 

**Figure 7:**
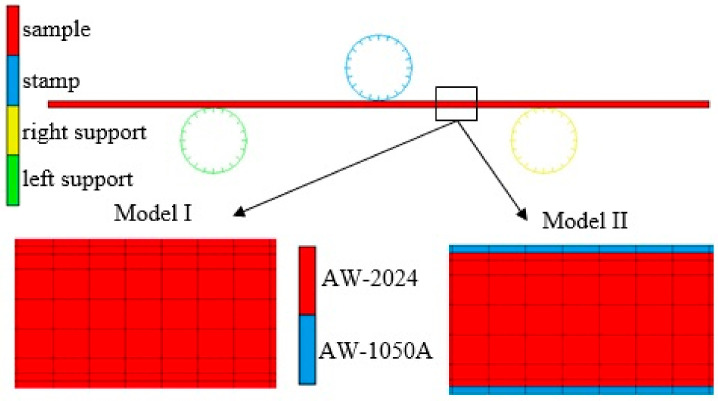
Numerical models of the bending process: sample 2D model (**top**), Model I without considering the material properties of the clad layers (**bottom left**), Model II taking into account the material properties of the clad layers (**bottom right**).

**Figure 8:**
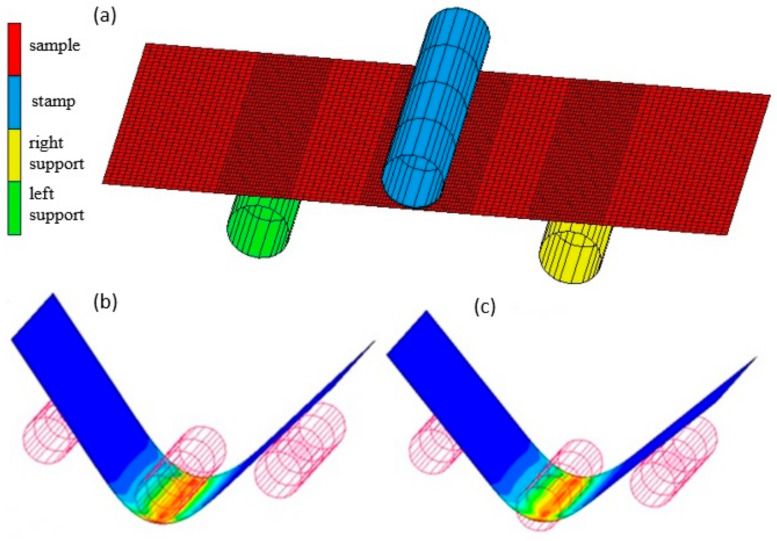
Sample 3D shell model: (**a**) FE mesh, (**b**) under load, and (**c**) unloaded.

The simulations of the bending process were carried out with the use of two 2D numerical models (Figure 7) and three 3D shell models (Figure 8): model I—a 2D model analysed in a plane deformation state disregarding the material properties of the clad layers (Figure 7, left), model II—a 2D model analysed in a plane deformation state taking into account the material properties of the clad layers (Figure 7, right), model III—a 3D shell model with the Huber–von Mises isotropic plasticity condition, model IV—a 3D shell model with the Hill anisotropic plasticity condition, V model—a 3D shell model with the Barlat anisotropic plasticity condition. For model II, rigid connections between the cladding and the base material were assumed. The cladding material properties were defined for the outer layers of the mesh. For models III–V, due to the inability to use shell elements in tasks requiring two-sided contact [22], the different material properties of the cladding layers over the sheet were not taken into account. The process of unloading the sample was modelled by switching off the contact between the sample and the bending punch, which caused springback to occur.

For the discretization of the deformable body in 2D models, quad 4 type 11 elements were used. Element type 11 is a four-node, isoparametric, arbitrary quadrilateral written for plane strain applications. For these elements, a modified interpolation scheme was used to improve the bending characteristics of the elements. This allows the ability to capture pure bending using a single element through the thickness. This substantially improves the accuracy of the solution, although the stiffness assembly computational costs increase [22]. The finite element meshes in model I and model II were the same. Four hundred finite elements were used along the length and nine finite elements were used in the thickness of the bent sample (Figure 7). The isotropic Huber–von Mises plasticity condition was used in 2D models. In contrast to discretization of the deformable body in the 3D shell models, 4-node quad 4 bilinear elements of type 75 were used in the formula, considering the effects of transverse shear and improving the behaviour of the shell elements during bending [20]. For these shell elements, the number of integration points on the thickness was 11. The finite element meshes in all 3D shell models were the same (Figure 8a). The size of the mesh elements was 1 mm in the width and length of the bent sample. Additionally, the finite element mesh along the length of the bent specimen in the areas of contact with the tools was densified and amounted to 0.5 mm. Ultimately, each of the 3D shell models consisted of 5120 finite elements. In order to make the results of numerical calculations independent of the mesh size of bodies modelled as deformable, during the development of the FEM model, the influence of the finite element ship size was analysed by densifying the mesh. After each compaction of the mesh, the bending load course was compared with the course of this force obtained with the use of the mesh before refinement. In this way, the nets were compacted until their further compaction had no greater impact on the compared parameter. The material parameters used to calculate the required coefficients in the Hill [23] and Barlat [24] models are shown in Table 1. Additionally, the value of the exponent m in the Barlat equation was assumed to be m = 8 [24].

## 4. Results and Discussion

In order to describe the material properties of a deformable sheet, an elastic plastic material model with nonlinear strain hardening was adopted. The material parameters of the tested sheets and clad layer were determined experimentally based on a uniaxial tensile test for four models of strain hardening. The material constants in Equations (2)–(5) were determined for the individual strain hardening curves using the least squares method by applying the Logger Pro program. The degree of matching of the individual equations of the strain hardening curve to its course obtained during the tensile test was assessed using the B_f_ index, which was calculated by relating the root mean square error, RMSE, to the mean feature level of σ_p_:

(10) $$B_{f}=\frac{\mathrm{RMSE}}{{\left(\sigma_{p}\right)}_{\mathrm{av}}}100\%$$

The values of material parameters in models (2)–(5) and the values of the fit error (Table 2) ware calculated as the average of the three sample orientations with respect to the rolling direction from the formula:

(11) $$X_{\mathrm{av}}=\frac{\left(X_{0}+2X_{45}+X_{90}\right)}{4}$$

where X—the value of selected material parameter, the subscripts denote the orientation of the specimen with respect to the rolling direction of the sheet.

**Figure 9:**
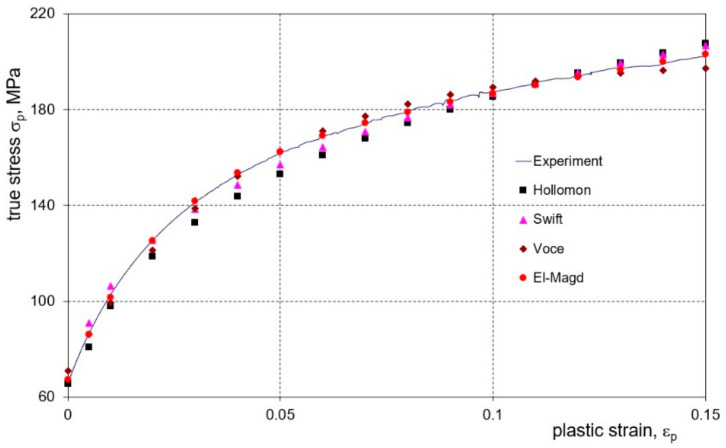
The degree of fitting of the experimental strain hardening curve for a 1 mm thick sheet in the direction 0° using Equations (2)–(5).

A comparison of the values of errors in fitting the strain hardening curves with the use of individual constitutive models shows that the El-Magd model was the best for both the sheet material and the clad layer material (Table 5). For example, for a 1.0 mm thick sheet, the error of fit with the El-Magd model was approximately 5 times smaller than for the Hollomon model and approximately 4 times smaller than for the Swift and Voce models. Even greater differences were found in the case of the 2.0 mm thick sheet metal. The best degree of fit of the El-Magd model to the results of the experiment in the entire range of sample elongation is also documented in Figure 9. Additionally, it should be noted that the degree of matching of individual Equations (2)–(5) was estimated for the range of tensile test deformation was close to the range of deformations observed in the bending process.

**Figure 10:**
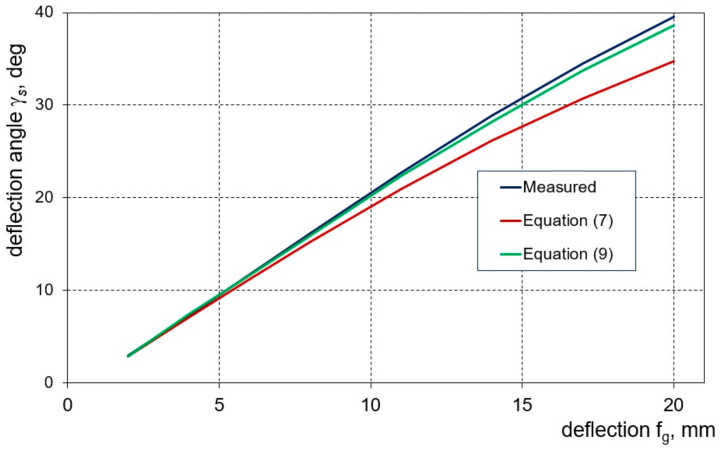
Comparison of the measured values of the sample deflection angle with the results of calculations according to Equations (7) and (9).

In order to assess the validity of introducing the relationships (8) and (9) to determine the sample deflection angles, a comparison of the values of the deflection angles measured for the samples after springback, γ_s_, with the results of the angles calculated based on the deflection arrow value was conducted. On the basis of the obtained characteristics (Figure 10), it can be seen that the values of the bending angles calculated with the use of Equation (9) are definitely closer to the values determined by the measurements than those calculated using Equation (7), especially for larger deflection values of the samples.

**Figure 11:**
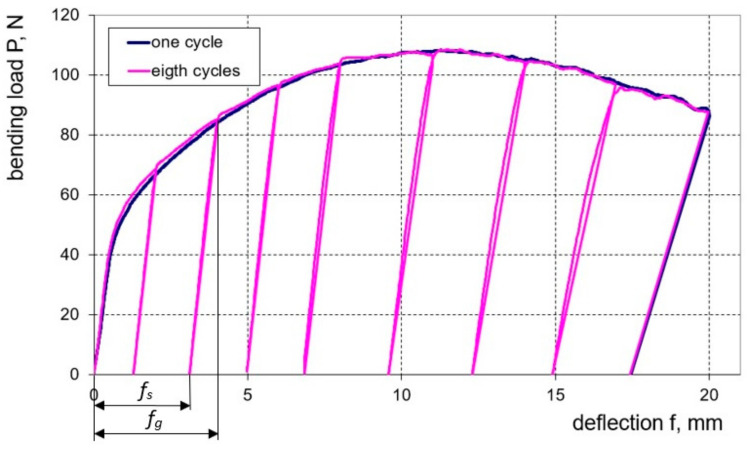
Characteristics of the bending load as a function of the deflection arrow for one load cycle (blue line) and eight load cycles (pink line), for the example of a 1.0 mm thick plate.

As mentioned earlier, in order to determine the dependence of the bending load on the deflection value, tests were carried out according to two schemes: multi-cycle bending with sample unloading and single-cycle bending. It is worth noting here that during bending, the surface of the samples did not reveal any changes that could be caused by damage to the clad layer. Based on the bending characteristics obtained (Figure 11), it can be seen that for both sheet thicknesses, the curves of single-cycle and multi-cycle bending practically coincide with each other. Such bending characteristics can be useful in fairly common cases when to obtain the desired curvature of the finished product it is necessary to re-bend it to compensate for springback. The numerical values of the band deflection arrows f_g_ and f_s_ both in the experiment and in the modelling were determined on the basis of the measurement points of the bending characteristics for eight load cycles of the sample. The method of reading the band deflection arrows f_g_ and f_s_ for the example of the second load cycle of the sample is shown for the load characteristic in Figure 11. The values of deflection arrows f_g_ and f_s_ determined experimentally and calculated from individual numerical models are presented in Table 6 for a sheet of 1 mm thickness and Table 7 for sheet metal of 2 mm thickness. 

**Figure 12:**
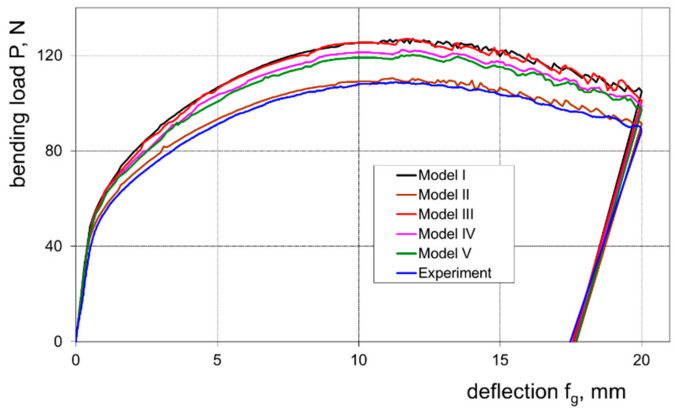
Experimentally and numerically calculated courses of the bending load as a function of the deflection arrow for a 1.0 mm thick sheet.

**Figure 13:**
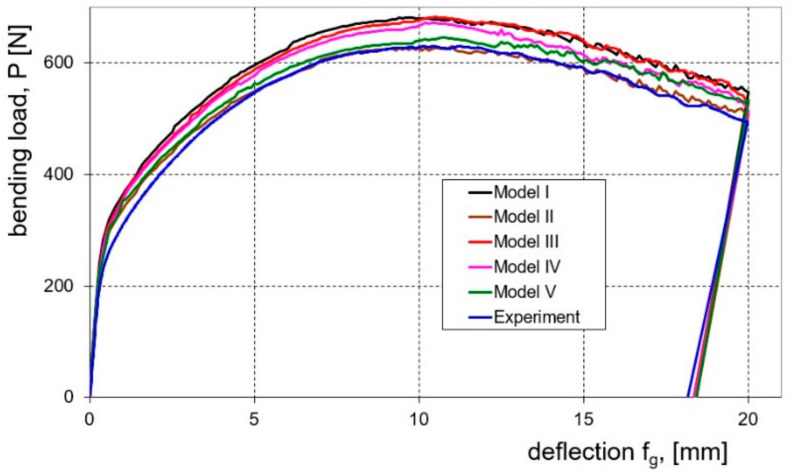
Experimentally and numerically calculated courses of the bending load as a function of the deflection arrow for a 2.0 mm thick sheet.

On the basis of the conducted research, the influence of the investigated numerical models on the effectiveness of prediction of springback and the course of the bending load characteristics was determined. Graphs (Figure 12 and Figure 13) show the bending load courses determined experimentally and calculated for the five numerical models tested, respectively, for sheets with thicknesses of 1.0 and 2.0 mm. These results indicate that the bending load characteristic determined numerically with the use of model II is the closest to the bending load characteristic determined experimentally. In order to estimate which of the proposed numerical models I–V is most useful for determining the value of the bending load, the relative error value of the maximum bending load was determined based on measurements and numerical calculations according to the relationship:

(12) $$B_{r}=\frac{\left|X_{\mathrm{EXP}}-X_{\mathrm{FEM}}\right|}{\left|X_{\mathrm{EXP}}\right|}100\%$$

where X_EXP_ and X_FEM_ are the maximum values of the analysed parameter determined experimentally and numerically, respectively, with the use of various constitutive equations.

**Figure 14:**
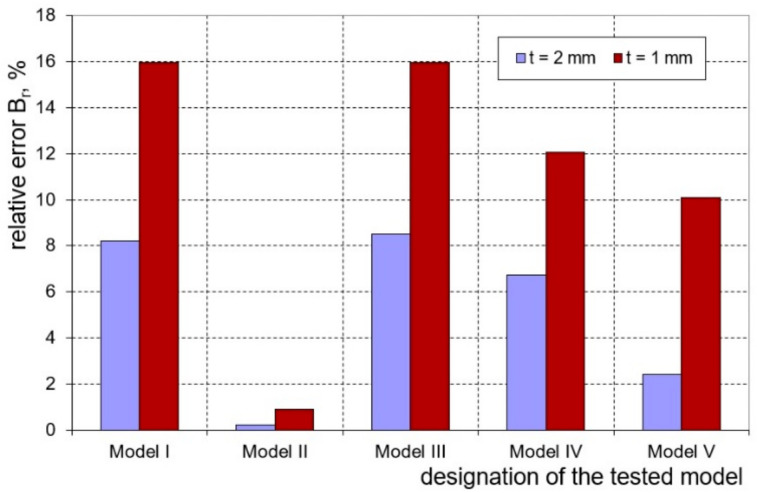
Comparison of the relative calculation error of the maximum bending load for the tested sheets depending on the numerical models used.

In the case of both tested sheets, the best agreement of the numerical modelling results with the results of experimental measurements was found for model II, which takes into account the presence of the cladding layers (Figure 14). The observed higher values of determined error for the sheet with a thickness of 1.0 mm according to all applied numerical models result from the fact that for this sheet the percentage of cladding layer thickness is almost twice as high as for the sheet with a thickness of 2.0 mm.

**Figure 15:**
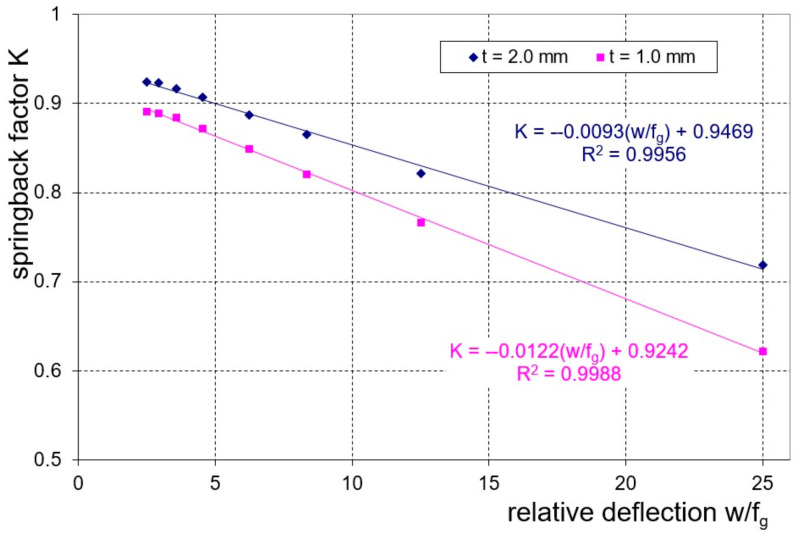
Experimental springback characteristics of the tested sheets.

On the basis of the measurement of the amount of elastic deformation of the tested samples, the springback characteristics were determined. The springback characteristics are presented as the dependence of the springback factor, K, on the relative deflection, w/f_g_ (w—distance between supports and f_g_—deflection arrow under load). In the case of both tested sheets, we obtained linear relationships with a high degree of correlation (Figure 15).

**Figure 16:**
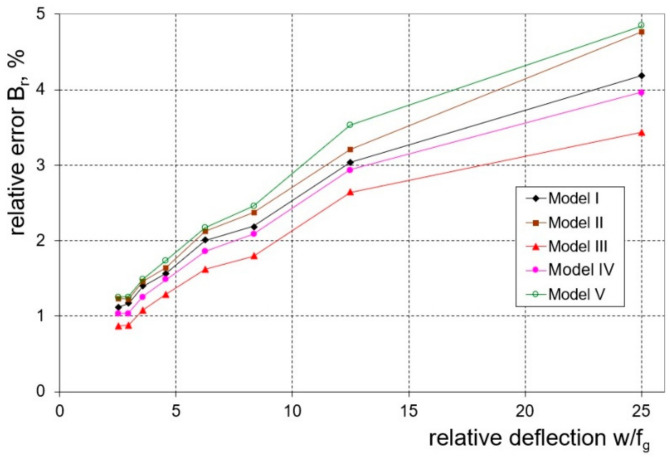
The values of the relative error in the calculation of the springback factor, K, of a 2.0 mm thick sheet depending on the quotient (w/f_g_) for the tested FEM models.

The next step in the research was to determine which of the analysed numerical models correlated best with the results of the experimental measurements. To assess the correctness of the models used, the value of the relative error determined according to Equation (12) was used, as in the case of the bending load. In the case of springback characteristics, Model III (3D shell model with the Huber–von Mises isotropic plasticity condition) can be considered as the best numerical model (Figure 16). The values of the relative error for the entire range of bending curvatures of the 1.0 mm thick sheet metal ranged from 0.96 to 3.24%, while for the 2.0 mm sheet it ranged from 0.87 to 3.44%. The numerical model II (which takes into account the presence of the cladding layers), which gave the best degree of fit to the experimental results in the case of the bending load, showed a slightly lower fit in the case of springback characteristics, but the relative error values were still within the acceptable range. For this model, the values of the relative error for the entire range of bending curvatures of the 1.0 mm thick sheet metal ranged from 1.54 to 5.49%, while for the 2.0 mm sheet it ranged from 1.25 to 4.77%.

## 5. Conclusions

This work presents the results of an analysis of the bending load and the springback phenomenon observed during elastic plastic cold shaping of AW-2024 aluminium alloy with AW-1050A aluminium cladding. The main conclusions of this work can be summarized as follows:

- Among the four popular models of yield stress, the El-Magd model proved to be the most suitable for describing the course of the material hardening curve of the tested sheets, for which the average error of the curve fitting was approx. 0.5%, while for the Hollomon model it was about 3%.
- Among the five numerical models tested, Model II, taking into account the cladding layer, turned out to be the most effective in forecasting the bending load, for which the relative error value was below 1% regardless of the tested sheet.
- For all the tested models, a significant influence of the ratio of the thickness of the cladding layer to the thickness of the sheet on the accuracy of the calculated bending load was found. With the increase in the proportion of the cladding layer in the sheet thickness, the accuracy of bending load prediction decreases. In the tested case, an increase in the thickness of the cladding layer from 5.5% to 10% of the sheet thickness resulted in an approximately two-fold increase in the relative error of the maximum bending load for all models, except for Model V, where the increase was approximately four-fold.
- The developed method for calculating the bending angle on the basis of the deflection arrow is much more accurate than the previously known one, as it additionally takes into account the influence of the tool radii and the thickness of the bent strand on the bending angle value.
- Out of all the numerical models tested, Model III turned out to be the most effective in predicting the amount of springback after bending.

## Figures and Tables

**Table 1:** Chemical composition of tested sheet material [14].

Si	Fe	Cu	Mn	Mg	Cr	Zn	Ti	Zr + Ti	Others		Aluminium Min.
									Each	Total	
≤0.5	≤0.5	3.8–4.9	0.3–0.9	1.2–1.8	≤0.1	≤0.25	≤0.15	≤0.2	0.05	0.15	Remainder

**Table 2:** Selected material parameters of the tested sheet material.

Direction of Sampling	Yield Stress σ_p0.2,_ MPa			Lankford Normal Anisotropy Coefficient r		
	Sheet 1.0 mm	Sheet 2.0 mm	Clad Material	Sheet 1.0 mm	Sheet 2.0 mm	Clad Material
0	66.89	74.89	28.92	0.728	0.679	1.03
45	63.43	72.09	30.07	0.674	0.728	0.79
90	64.73	72.95	31.22	0.581	0.678	1.24
Mean value	64.62	73.01	30.07	0.664	0.703	0.96

**Table 3:** Data for determining the coefficient A for w = 50 mm.

f_g_ or f_s_ mm	γ_g3_ or γ_s3_*,* rad			
	t/r			
	0.1	0.16	0.2	0.4
0	0	0	0	0
2	0.0812	0.0812	0.0813	0.0814
4	0.1643	0.1644	0.1646	0.1651
6	0.2481	0.2485	0.2488	0.2501
8	0.3315	0.3323	0.3327	0.3351
11	0.4534	0.4548	0.4557	0.4602
14	0.5682	0.5702	0.5716	0.5787
17	0.6731	0.6759	0.6777	0.6875
20	0.7667	0.7701	0.7725	0.7845
A	0.4304	0.4446	0.4541	0.5025
RMSE	8.2 × 10^−4^	8.4 × 10^−4^	8.4 × 10^−4^	7.6 × 10^−4^

**Table 4:** Data for determining the coefficient A for w = 100 mm.

f_g_ or f_s_ mm	γ_g3_ or γ_s3_*,* rad			
	t/r			
	0.1	0.16	0.2	0.4
0	0	0	0	0
2	0.0402	0.0402	0.0402	0.0402
4	0.0805	0.0805	0.0806	0.0806
6	0.1210	0.1210	0.1211	0.1212
8	0.1614	0.1614	0.1615	0.1617
11	0.2216	0.2217	0.2218	0.2223
14	0.2810	0.2812	0.2814	0.2821
17	0.3391	0.3394	0.3397	0.3408
20	0.3956	0.3961	0.3964	0.3979
A	0.2119	0.2178	0.2225	0.2421
RMSE	5.1 × 10^−6^	2.6 × 10^−5^	4.3 × 10^−5^	2.6 × 10^−5^

**Table 5:** The results of the evaluation of the error of fitting the individual Equations (2)–(5) of the strain hardening curves to the course of the experimental curve.

Strain Hardening Curve Model	Designation of Material Constants	Average Values from Three Directions (0°, 45° and 90°)					
		1.0 mm Sheet		2.0 mm Sheet		Cladding Material	
		Value of the Constants	Fit Error B_f_, %	Value of the Constants	Fit Error B_f_, %	Value of the Constants	Fit Error B_f_, %
Hollomon	K_1_, MPa	325.78	2.725	352.99	3.375	114.6	1.138
	n_1_	0.268		0.256		0.225	
Swift	K_2_, MPa	310.3	2.032	332.42	2.571	115.5	0.845
	ɛ _0_	0.00090		0.00059		0.00320	
	n_2_(t)	0.244		0.23		0.2329	
Voce	A_3_, MPa	71.01	2.042	76.85	1.803	34.98	2.933
	K_3_, MPa	122.12		123.22		51.56	
	n_3_	23.31		32.17		10.76	
El-Magd	A_4_, MPa	66.47	0.519	72.22	0.240	31.60	0.464
	B_4_, MPa	277.15		301.32		95.12	
	K_4_, MPa	84.83		92.31		28.61	
	n_4_	42.00		52.26		33.84	

**Table 6:** Experimental and calculated values of the deflection arrow f_s_ for given values of f_g_ for a 1 mm thick sheet.

f_g_ mm	f_s,_ mm					
	Experiment	Numerical Model Designation				
		I	II	III	IV	V
2	1.251	1.298	1.347	1.291	1.311	1.319
4	3.080	3.150	3.201	3.126	3.151	3.162
6	4.939	5.025	5.079	5.012	5.043	5.056
8	6.800	6.903	6.962	6.887	6.925	6.941
11	9.553	9.689	9.754	9.669	9.713	9.731
14	12.259	12.418	12.494	12.394	12.449	12.471
17	14.870	15.059	15.140	15.029	15.094	15.119
20	17.359	17.594	17.700	17.558	17.637	17.677

**Table 7:** Experimental and calculated values of the deflection arrow f_s_ for given values of f_g_ for a 2 mm thick sheet.

f_g_ mm	f_s,_ mm					
	Experiment	Numerical Model Designation				
		I	II	III	IV	V
2	1.444	1.504	1.512	1.493	1.501	1.513
4	3.301	3.399	3.405	3.387	3.396	3.415
6	5.205	5.317	5.326	5.297	5.311	5.330
8	7.103	7.243	7.252	7.217	7.233	7.255
11	9.956	10.114	10.121	10.086	10.105	10.131
14	12.758	12.947	12.955	12.904	12.928	12.959
17	15.520	15.723	15.733	15.673	15.701	15.739
20	18.166	18.411	18.436	18.355	18.392	18.440

## Data Availability

Not applicable.
